# Supplementary material for: Patterns of Intron Gain and Loss in Fungi
Source: PLoS Biol. 2004 Nov 30;2(12):e422. doi: 10.1371/journal.pbio.0020422 (PMC532390; doi:10.1371/journal.pbio.0020422)
Supplement: Table S1 — Also available at http://genes.mit.edu/NielsenEtAl/. (4.3 MB ZIP). [file pbio.0020422.st001.zip › NielsenEtAl/html/551.html]

AN3594.1.NCU04137.1.MG06743.1.FG02011.1


```
 CLUSTAL W (1.82) Multiple Sequence Alignments - Introns Inserted


Sequence 1: NCU04137.1	606 aa
Sequence 2: FG02011.1	558 aa
Sequence 3: MG06743.1	607 aa
Sequence 4: AN3594.1	561 aa
Alignment Length: 647 aa
Number Identitical Residues: 290 aa
Alignment Score (without introns) 13651


MG06743.1 	MDP-LEEAPWAD~AATTSTSQ-SSSQQHQDDNTATSKPSESSAGGSSSLS-ASTGGPRPS
NCU04137.1	-----MEDPWAD~SAIGGESNNSSSTVPQSDDTAPADSTAAPSTSSTLTPSANTSSSRPS
FG02011.1 	MD--VEESPWAD~SSQ------------ASQQASQPEASASASQSSTPAP-ASSSAPRPS
AN3594.1  	MDLDGGDSPWGD1VPS--------------QSTNNLAASQSETEGTGAFP---PSLTLMC
          	 . .  : **.*  .               :.:    .: : : .:   .   .. .  .

MG06743.1 	RLTPRRLLAQPTRLQAVEDDPLG-~PLGAPPSTGATTPGAAPLSENLSPTGGSSDTGPPA
NCU04137.1	RLTPRRLVAQPTRLEAVKDDPLG-~PLGAST-----------------PTATDTPLAPPQ
FG02011.1 	RG-PRRLVAQPTRLEAV-DDPLG-~PLGAVP-----------------EDNGSAQDAPPV
AN3594.1  	GLMLTPTLRSPRPINTARDTPFDR1PLGDKP----------------AENSPTGLEQAPV
          	       : .*  :::. * *:.  ***  .                :         .* 

MG06743.1 	PPKEHSLAIR---PGGGAQQ----RR--GGPPDPHRIDDDDDDDRLFDTTNRGPRVPPPV
NCU04137.1	PPLKEQLPLRTTLSGGPNQIGSGARRPGGGPSDPHRIEEEEL----YNDSSSGPRQPPPV
FG02011.1 	PPQKEQMVIRTIMSQ-PQQQ----RR----AADPNHIDDEE------NDGPKAPRAPPPV
AN3594.1  	PPQKEAFASRNVRPASTTSQT---------SSGAGMMDSVN-----LEEDGTGFRNPPPV
          	** :. :  *.  . .  .           ....  ::. :      :    . * ****

MG06743.1 	APAPHGPGGAARPGGVAQSVSIEQAAKPTFWITVGDPHKVGDLTSSHIVYSVRT------
NCU04137.1	PPALPSPVRTS----MQPSVSIEQAANPTFHISVGDPHKVGDLTSSHIVYSVRTKVRNVE
FG02011.1 	EAAQPSAVRSS----NQPSVSIEQAAKPTFQIYVGDPHKVGDLTSSHIVYSVRT------
AN3594.1  	QPPSDVEASKRQ---QQPSVSVEKAAHPTFEISVGDPHKVGDLTSSHIVYQVRT------
          	 ..               ***:*:**:*** * *****************.***      

MG06743.1 	--------------------K0TTSKGYKQPEFEVKRRYRDFLWLYNTLHGNNPGVVVPP
NCU04137.1	IPSYSPIFGTTDVLTTTEQLK~TTSKAYKQPEFEVKRRYRDFLWLYNTLHANNPGVVVPP
FG02011.1 	--------------------K0TTSKGYKQPEFEVKRRYRDFLWLYNTLHGNNPGIVVPP
AN3594.1  	--------------------K0TTSKAYRQPEFTVSRRYRDFLWLYNSLHSNNPGVVVPP
          	                    * ****.*:**** *.***********:**.****:****

MG06743.1 	PPEKQAVGRFESNFVESRRQALEKMLNKIAVHPTLQHDPDLKLFLESESFNVDIKHKERR
NCU04137.1	PPEKQAVGRFESNFVEARRAALEKMLNKTAAHPTLQHDADLKLFLESEAFNIDVKHKERK
FG02011.1 	PPDKQAVGRFDSNFVESRRAALEKMLNKTAIHPTLQHDPDLKLFLESETFNVDIKHKERR
AN3594.1  	PPEKQAVGRFDTNFVESRRAALERMLNKIAAHPILQHDGDLKIFLESEAFNVDIKNKENR
          	**:*******::****:** ***:**** * ** **** ***:*****:**:*:*:**.:

MG06743.1 	EPLNMVGGESKGMFSSLGIG-GSTNKFVEQDD0WFHERRVYLDAMENQLKALLKAMETMV
NCU04137.1	EPH---LGESKGVLSTFGISVGSGNKFVEQDD0WFHDRRVYLDALENQLKGLLKAMDSMV
FG02011.1 	DPI---PTESKGVLGSLGINVGGGNKFVEQDD~WFHDRKVYLDALENQLKGLLKAMETMV
AN3594.1  	EPD---LGQSKGMFSSFGISVGGGGKFIEHDD0WFHDRKIYLDALENQLKSLMKAIDTVV
          	:*      :***::.::**. *. .**:*:** ***:*::****:*****.*:**::::*

MG06743.1 	GQRKAMAEAAGDFSASLHALSTVELSPALSGPLDALSELQTTIQDVYDRQAQQDVLTFGI
NCU04137.1	AQRKAMAEAAGEFSASLHALSTVELSPTLSGPLDALSELQLTIRDVYDRQAQQDVLTFGI
FG02011.1 	GQRKMMAEAAGDFSASLHALSTVELSPSLSGPLDALSELQLTIRDVYDRQAQQDVLTFGI
AN3594.1  	AQRKGLSEAAGEFSTSIQSLAAVELSPLLSGPLYGLSDLQLRIKELYDRQAQQDVLTLGI
          	.*** ::****:**:*:::*::***** ***** .**:**  *:::***********:**

MG06743.1 	VIEEYLRLIGSVKQAFAQRQKAFYAWHGAETELNKRRAAQDKLLRQGRSQQDRLNHVSAE
NCU04137.1	IIEEYIRLIGSVKQAFSQRQKAFYSWHSAESELQKRKASQDKLLRQGKSQQDRLNQVNAE
FG02011.1 	ILEEYIRLIGSVKMAFGQRQKAFYSWHAAESELQKKKATQDKLLRQGKSQQDRLNQMSAE
AN3594.1  	TIDEYLRLIGSVKTAFSQRQKAYHSWHAAESELQKRKHSQEKLLRQGKSQQDRLNQANAD
          	 ::**:******* **.*****:::**.**:**:*:: :*:******:*******: .*:

MG06743.1 	VADAERKVHQARLLFDDMGKLMRTELDRFEREKVEDFKSGVETFLESAIEAQKE0LIEKW
NCU04137.1	VADAERKVHQARLLFEDMGRLMRAELDRFEREKVEDFKSGVETFLESAVEAQKE0LIEKW
FG02011.1 	VGESERKVHQARLLFEDMGRLMRSELDRFEKEKVEDFKSGVETFLESAVEAQKE0LIEKW
AN3594.1  	VADAERRVHQARLLFEDMGRLMRNELQRFEKEKVEDFKSGVETFLESAVEAQKE0LIELW
          	*.::**:********:***:*** **:***:*****************:***** *** *

MG06743.1 	ETFLMQLDADDDDAAFYKPPVLASADNGGGNNKPAGQTAVDRARARIDEDSD
NCU04137.1	ETFLMQLDAEDDETVFYRPPVVQAN-------KPAGDTAVDRARARIDDDSD
FG02011.1 	ETFLMQLDAEDDESVFYRPPVYQQQQ------QKGGDTAVDRARARIDEDSD
AN3594.1  	ETFLLQLDAGEDGNPFYPPAGEHNAS------AESAPGAPSESTVSTAEAA-
          	****:**** :*   ** *.     .        ..  * ..: .   : :
```
